# What Is the Matter with American Veterinary Journalism?

**Published:** 1895-08

**Authors:** 


### What is the Matter with American Veterinary Journalism

For one year, with this number, the managing editor, with his associate editors, has given assiduous attention to the building-up of the Journal in a broader journalistic sense than was attempted before for several years. A strong appeal has been earnestly made to the veterinary profession for a more generous support than has been given in the past, with the one primary aim and object, that the Journal should become self-supporting. We have placed from month to month an extra edition in the hands of every known veterinarian outside of our subscription list in the United States and in parts of Canada; we have added new features to the Journal; exceeded for a number of months the regular number of pages, with valuable, interesting, and instructive contributions pertaining to a wide variety of subjects, and yet we are forced to believe that we have not yet made the Journal what is needed by the profession. We say this because there are to-day in North America at least five thousand veterinarians who depend entirely, or in great part, on the work of the profession for a livelihood, and of this number we believe, from a careful and thorough covering of the field, that not more than fifty per cent. take an American veterinary journal. We have asked ourselves why, and have tried to answer by the constant addition of new features, and yet we feel at this time like asking of those of the profession who are not readers of veterinary journals, How can you get along in your labors without a knowledge of what your fellow-members of the craft are doing, what the world is doing in your particular sphere of work, and what is the matter with American veterinary journalism?

---

THE veterinarians of Wisconsin should not forget the importance of their State meeting at Chippewa Falls on the 14th of August. Besides the pleasure from a closer social union of the profession a very interesting programme has been provided for their entertainment and instruction. The near approach of the National Convention at Des Moines, Iowa, on September 10th, 11th, and 12th, should also be a matter for consideration, and delegates should be chosen to represent the Association at the national meeting.

